# Case report: Paroxysmal nocturnal hemoglobinuria presenting with hemorrhagic esophageal varices

**DOI:** 10.3389/fmed.2023.1276030

**Published:** 2023-10-25

**Authors:** Runsen Du, Lihua Zheng, Peng Liu, Yaheng Zhao, Yan Yang, Lei Zhang, Zengren Zhao

**Affiliations:** ^1^Department of Gastrointestinal Surgery, The First Hospital of Hebei Medical University, Hebei, China; ^2^Department of Vascular Surgery, The First Hospital of Hebei Medical University, Hebei, China

**Keywords:** paroxysmal nocturnal hemoglobinuria, portal vein thrombosis, esophageal varices, hemorrhagic esophageal varices, TIPS

## Abstract

We report the case of a female who was cured of hemorrhagic esophageal varices caused by paroxysmal nocturnal hemoglobinuria (PNH) through transjugular intrahepatic portosystemic shunt (TIPS) treatment. PNH complicated by portal vein and visceral veins thrombosis without hepatic veins is extremely rare, and as such, it is easy to incorrectly treat due to lack of awareness. Hemorrhagic esophageal varices due to PNH with PVT have been reported in one case in 1974, and here, we report the second.

## 1. Introduction

Paroxysmal nocturnal hemoglobinuria (PNH) is a rare type of thrombophilia and hematopoietic stem cell disorder (1). Thrombophilia of PNH has been defined as “the most vicious acquired thrombophilic state known to medicine,” representing the leading cause of death in PNH patients (2). PNH with portal vein thrombosis (PVT) without hepatic vein thrombosis has been sparsely reported, with several cases of esophageal varices and only one case of esophageal variceal bleeding (3). The pathophysiology of how PNH causes PVT specifically is still unclear. PVT was once considered a contraindication for transjugular intrahepatic portosystemic shunts (TIPS). In recent years, there has been an increasing amount of literature discussing how patients with PVT may benefit from TIPS to reduce portal venous pressure and complications such as hemorrhagic esophageal varices (4). Long-term portal vein thrombosis can cause cavernous transformation of the portal vein (CTPV). Whether TIPS is used in non-cirrhotic portal vein cavernous transformation is controversial (5). Here, the case of a patient with CTPV and PVT caused by PNH is presented.

## 2. Case presentation

A 49 years-old female presented to the vascular surgery department of our hospital with melena that had lasted for 18 months. While 18 months prior to this the patient had a little intermittent melena, which resolved with conservative treatment, the symptoms suddenly worsened 6 months before presenting to our hospital after a CT examination at her local hospital revealed esophageal and gastric varices and splenomegaly; she underwent selective splenic artery embolization (SSAE) to relieve hypersplenism and portal vein pressure. After SSAE, her hemoglobin changed from 6.6 to 5.7 g/dL, and her platelet changed from 28 to 19 × 103/μL. Then, she was discharged from the hospital. However, 5 months after the SSAE, melena reappeared more severely than before. No other gastrointestinal symptoms, such as nausea, vomiting, or abdominal pain, were present during the onset of the disease.

The patient reported symptoms of anemia, leukopenia, and thrombocytopenia 9 years ago and also reported a 9 years history of PNH confirmed by flow cytometry to identify GPI-AP-deficient peripheral blood cells. During routine physical exams 5 years before, contrast-enhanced CT reported PVT, but splenomegaly and varicose veins were not reported. The patient did not show any PVT-related symptoms. She had taken prednisolone and warfarin and had no history of surgical intervention. She had no family history of thrombosis.

The patient’s temperature was 36.5°C, heart rate was 92 beats per minute, respiratory rate was 18 breaths per minute, and blood pressure (measured with an electronic cuff) was 86/50 mmHg. The physical examination showed an anemic appearance, splenomegaly, and tenderness on the left upper quadrant.

A complete blood count revealed a leukocyte count of 4.2 × 10^9^/L, a hemoglobin count of 4.7 g/dL, and a platelet count of 29 × 10^9^/L. Her coagulation test showed a D-dimer of 7.63 U/L and fibrinogen degradation products of 33.87 mg/L. She presented with a lactate dehydrogenase (LDH) level of 3,657 IU/L (reference range, 120–250) as a result of hemolysis. A bone marrow smear revealed significant hyperactivity of hematopoiesis, especially in the erythroid lineage.

**Figure 1:**
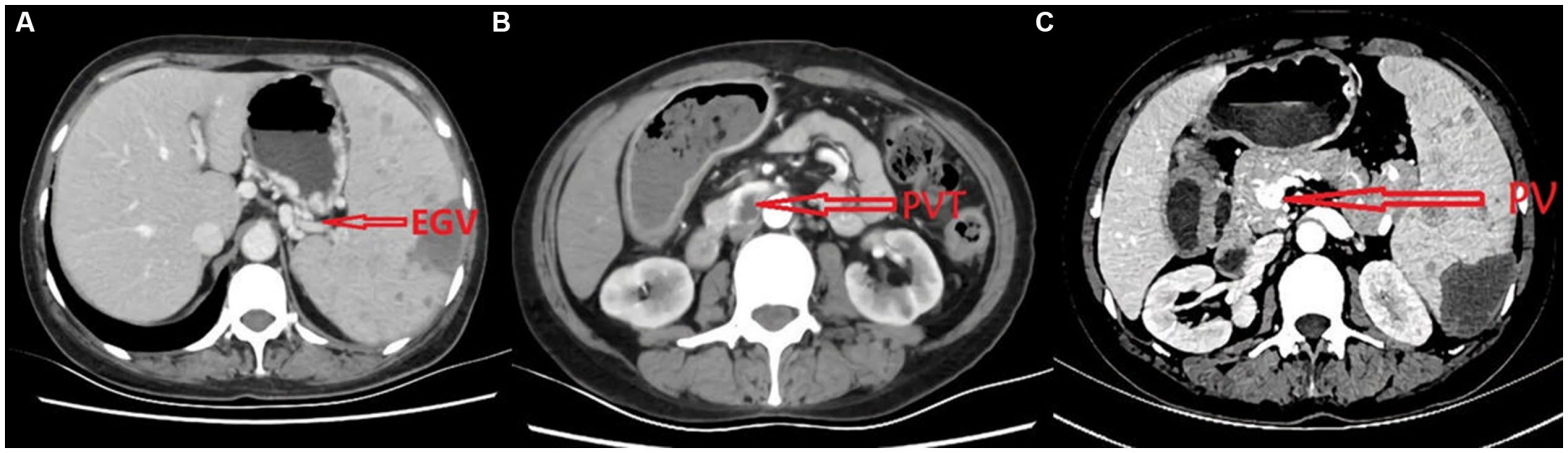
The CT scan before SSAE **(A)**, before TIPS **(B)**, and after TIPS **(C)**.

Computed tomography (CT) scan results from the local hospital 5 months before showed revealing esophageal and gastric varices (EGV) before SSAE (Figure 1A), splenomegaly (18.8 cm), and wider portal vein diameter. A contrast-enhanced CT scan at our hospital revealed portal vein, splenic vein, and superior mesenteric vein thrombosis with extensive collaterals, including the esophageal and gastric varices and CTPV (Figure 1B). Moreover, the size of the spleen was 13.7 cm at this time.

The patient was diagnosed with PNH complicated by varices portal vein thrombosis and superior mesenteric vein thrombosis causing esophageal gastric vein bleeding.

**Figure 2:**
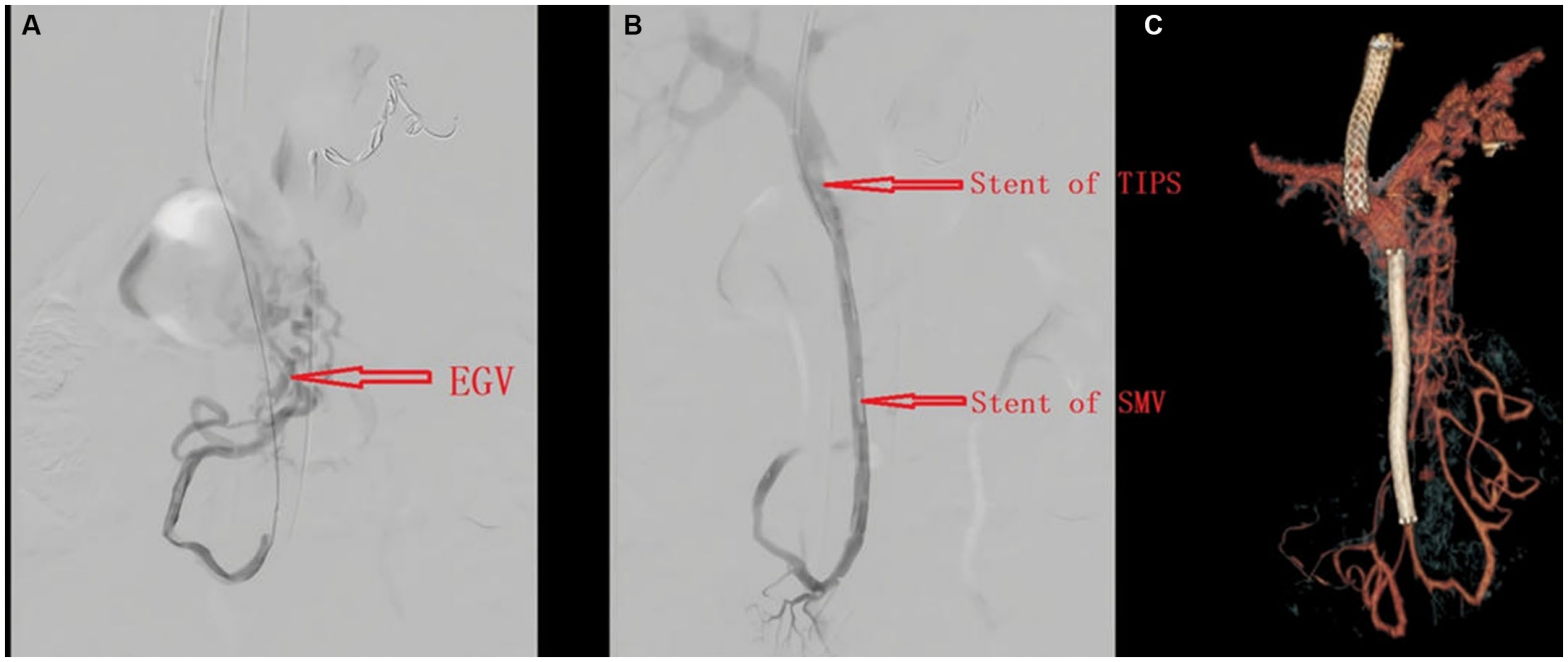
Angiography before **(A)** and after **(B)** TIPS placement; CT angiography after TIPS **(C)**.

Esophageal–gastric varices are visible through the imaging (Figure 2A). The patient underwent TIPS and superior mesenteric vein and splenic vein stent placement procedures (Figures 2B,C). She was asked to continue using prednisone. Intravenous heparin was administered, and she was transitioned to warfarin with a goal an international normalized ratio of 2.0 to 2.5.

The esophageal and gastric varices disappeared, and hemoglobin rose (Table 1). The patient no longer had blood in her stool, and her CT scans revealed smooth blood flow in the stents at her 1 month, 3 months, 6 months, and 1 year follow-up after the surgery (Figure 1C). There were no complications, such as hepatic encephalopathy, at the follow-ups.

**Table 1:** Changes in blood routine before and after TIPS.

Representative blood counts	Preoperative	POD 0	POD 1	POD 2	POD 3	POD 7	1-year follow-up
HGB (g/dL)	4.7	5.7	6	6.3	6.1	6.3	5.9
PLTs (×10^3^/μL)	29	46	25	27	23	17	34
Leukocyte (10^9^/L)	4.2	10.6	4.6	5.3	5	2.2	3.4
RBCs (10^12^/L)	1.35	1.7	1.82	1.86	1.82	1.79	1.8

**Table 2:** Previous similar case compared to ours.

Case	Age/sex	Chief complaint	Physical examination	HGB g/dL	LDH IU/L	Techniques	Thrombus site	Treatment	Ref.
1	37/female	Hematemesis and melena	Hepatosplenomegaly, anemic appearance	9	1,605	Endoscopy and angiogram	Splenic and portal vein	Portal-azygos disconnection, splenectomy, and pyloroplasty	(6)
Our case	49/female	Melena	Splenomegaly, anemic appearance and tenderness on left upper quadrant	4.7	3,657	CT and angiogram	Splenic, portal, and superior mesenteric vein	TIPS and superior mesenteric vein stent	

## 3. Discussion

PNH is a rare, acquired, potentially life-threatening hematologic disorder characterized by chronic intravascular hemolysis caused by uncontrolled activation of the terminal complement pathway (1). The mechanisms of thrombosis in PNH are still poorly understood; possible causes include the percentage of GPI protein-free granulocytes (PNH granulocytes >50%), endothelial cell damage, platelet activation because of the absence of CD59, and nitric oxide due to intravascular hemolysis, among others (7). Rother et al. (8) pointed out that thromboembolism is mainly due to hemolysis; although the mechanism is not fully understood, hemolysis has been implicated in the initiation of platelet activation and aggregation. Thrombotic events (TEs) account for up to 67% of deaths with a known cause in patients with PNH. Thrombosis can appear anywhere in the body (9). Hepatic veins are the most commonly involved site in PNH, and the isolated involvement of portal veins with visceral veins without the involvement of the hepatic veins is rare (10). PVT involving superior mesenteric veins and mesenteric venous arches may lead to intestinal ischemia, obstruction, and fatal intestinal infarction (11). PNH with PVT, on the other hand, is very rare and is easy to mistreat due to complex complications. A literature search of relevant articles on the PubMed database, published from January 1977 to May 2023, was conducted using the keywords “paroxysmal nocturnal hemoglobinuria” and “portal vein thrombosis.” One article presenting hemorrhagic esophageal varices due to PNH with PVT was identified (6).

The two cases are very similar, with both subjects being female and having experienced melena and splenomegaly (Table 2). In their case, hematemesis indicated that the patient may have bled faster and more than ours. In our case, hemoglobin was lower and LDH higher, possibly because of the more severe hemolysis caused by PNH. In addition, our patient presented with CTPV. In terms of treatment, their patient underwent portal vein dissection, splenectomy, and pyloroplasty. Our treatment modalities were more minimally invasive than their surgery and less invasive to the patient, gastric bleeding did not recur during follow-up, and hemoglobin recovered steadily.

There have also been several reports of PNH complicated by PVT and abdominal thrombosis (10) but with no bleeding of the esophageal varices. It is possible that our patient had not received effective treatment for a long time, and worsening portal hypertension led to hemorrhagic esophageal varices.

We conducted a thorough evaluation before treating the patient. The patient had completely blocked the portal vein and had previously undergone SSAE surgery for the treatment of regional portal hypertension. At this time, the patient developed refractory esophageal variceal bleeding and severe anemia, and we tried to save their life by using TIPS surgery to relieve portal hypertension. The right internal jugular vein was punctured for TIPS. The femoral vein was used as a puncture route for infarction of the superior mesenteric vein and portal vein, followed by a stent. Note that the superior mesenteric venous stent should completely cover the thrombus site while avoiding covering the main portal vein, and warfarin should be given to the patient after surgery.

The use of TIPS in PVT has been studied with the possibility of achieving recanalization by disrupting the thrombus and mechanical thrombectomy. The feasibility rate of performing TIPS in PVT ranges between 75% and 100% (12). CTPV was once considered a relative contraindication to TIPS, and with their attempts, some scholars believe that TIPS can be used for refractory CTPV. Currently, whether TIPS can be used in patients with CTPV, especially those without cirrhosis, is controversial (5). Our case is a deliberate and bold attempt.

We consider trilineage cytopenia in our patient primarily because of PNH instead of hypersplenism. Alleviating hypersplenism does not cure trilineage cytopenia, and hemorrhagic esophagus further aggravates anemia. After we performed TIPS on our patient and gave her warfarin and prednisolone, her bleeding was controlled, and the hemoglobin returned, but the levels were not very high because of PNH.

Our patient had spleen shrinkage after SSAE, but their thrombocytopenia did not improve. This outcome is different from that found by Araten et al. (13). We believe that SSAE not only has no value but may also have increased PVT. A CT scan before SSAE showed portal vein widening, suggesting decreased portal hypertension and reduced portal vein flow velocity and that SSAE results in splenic vein congestion that further reduces portal blood flow velocity, which may lead to PVT (14). In our case, portal vein recanalization and TIPS relieved portal vein pressure and cured hemorrhagic esophageal varices, and there were no complications, such as hepatic encephalopathy, at follow-up. TIPS and superior mesenteric vein stent have been shown to be successful in the treatment of hemorrhagic esophageal varices caused by PVT and superior mesenteric vein thrombosis in our PNH patient. Such treatments are effective in avoiding the worsening of the condition, including increased bleeding and intestinal necrosis.

Eculizumab has been found to be highly efficient in reducing intravascular hemolysis and may provide protective antithrombotic action (15). However, high costs and access difficulties have limited the utilization of eculizumab in China (16). Taking warfarin with a goal international normalized ratio of 2.0 to 3.0 is crucial for the unobstructed stent.

In our case, the patient’s initial visit was not at our institution, so we lacked some information, including upper endoscopy, etc., but by contrast-enhanced CT and the patient’s melena symptoms, we could also confirm the presence of esophageal and gastric variceal bleeding. Our brief report focuses on highlighting the improvement of melena and a steady recovery in hemoglobin after the patient received TIPS surgery. TIPS may become a new treatment option for PNH combined with PVT.

In conclusion, portal vein thrombosis in patients with paroxysmal nocturnal hemoglobinuria is rare and refractory to treatment. The long course of the disease facilitates portal cavernous transformation, and it is difficult to achieve a curative effect via simple spleen embolism. Our patient underwent transjugular intrahepatic portosystemic shunt, which worked well at 1 month, 3 months, 6 months, and 1 year follow-up and could inform clinical decision-making.

## Data availability statement

The original contributions presented in the study are included in the article/supplementary material, further inquiries can be directed to the corresponding author.

## Ethics statement

The studies involving humans were approved by the Ethics Committee of the First Hospital of Hebei Medical University. The studies were conducted in accordance with the local legislation and institutional requirements. The human samples used in this study were acquired from a by-product of routine care or industry. Written informed consent for participation was not required from the participants or the participants’ legal guardians/next of kin in accordance with the national legislation and institutional requirements. Written informed consent was obtained from the individual(s) for the publication of any potentially identifiable images or data included in this article.

## Author contributions

RD: Writing – original draft, Writing – review & editing. LiZ: Writing – review & editing, Formal Analysis, Funding acquisition. PL: Data curation. YZ: Investigation. YY: Validation. LeZ: Methodology, Conceptualization. ZZ: Conceptualization, Project administration, Supervision, Funding acquisition.
